# Caloric restriction reduces trabecular bone loss during aging and improves bone marrow adipocyte endocrine function in male mice

**DOI:** 10.3389/fendo.2024.1394263

**Published:** 2024-06-05

**Authors:** Charlotte Rinne, George A. Soultoukis, Masoome Oveisi, Marina Leer, Oskar Schmidt-Bleek, Lisa-Marie Burkhardt, Christian H. Bucher, Eman Abou Moussa, Melanie Makhlouf, Georg N. Duda, Luis R. Saraiva, Katharina Schmidt-Bleek, Tim J. Schulz

**Affiliations:** ^1^ Department of Adipocyte Development and Nutrition, German Institute of Human Nutrition Potsdam-Rehbrücke, Nuthetal, Germany; ^2^ German Center for Diabetes Research (DZD), München, Germany; ^3^ Julius Wolff Institute for Biomechanics and Musculoskeletal Regeneration, Berlin Institute of Health at Charité - Universitätsmedizin Berlin, Berlin, Germany; ^4^ Berlin Center for Advanced Therapies (BeCAT), Charité – Universitätsmedizin Berlin, Corporate Member of Freie Universität Berlin, Humboldt-Universität Berlin, Berlin, Germany; ^5^ Translation Medicine Division, Sidra Medicine, Doha, Qatar; ^6^ Berlin Institute of Health Centre for Regenerative Therapies (BCRT), Berlin Institute of Health at Charité - Universitätsmedizin Berlin, Berlin, Germany; ^7^ College of Health and Life Sciences, Hamad Bin Khalifa University, Doha, Qatar; ^8^ Institute of Nutritional Science, University of Potsdam, Nuthetal, Germany

**Keywords:** caloric restriction, aging, bone, bone marrow adipose tissue, osteogenesis

## Abstract

**Introduction:**

Caloric restriction (CR) is a nutritional intervention that increases life expectancy while lowering the risk for cardio-metabolic disease. Its effects on bone health, however, remain controversial. For instance, CR has been linked to increased accumulation of bone marrow adipose tissue (BMAT) in long bones, a process thought to elicit detrimental effects on bone. Qualitative differences have been reported in BMAT in relation to its specific anatomical localization, subdividing it into physiological and potentially pathological BMAT. We here examine the local impact of CR on bone composition, microstructure and its endocrine profile in the context of aging.

**Methods:**

Young and aged male C57Bl6J mice were subjected to CR for 8 weeks and were compared to age-matched littermates with free food access. We assessed bone microstructure and BMAT by micro-CT, bone fatty acid and transcriptomic profiles, and bone healing.

**Results:**

CR increased tibial BMAT accumulation and adipogenic gene expression. CR also resulted in elevated fatty acid desaturation in the proximal and mid-shaft regions of the tibia, thus more closely resembling the biochemical lipid profile of the distally located, physiological BMAT. In aged mice, CR attenuated trabecular bone loss, suggesting that CR may revert some aspects of age-related bone dysfunction. Cortical bone, however, was decreased in young mice on CR and remained reduced in aged mice, irrespective of dietary intervention. No negative effects of CR on bone regeneration were evident in either young or aged mice.

**Discussion:**

Our findings indicate that the timing of CR is critical and may exert detrimental effects on bone biology if administered during a phase of active skeletal growth. Conversely, CR exerts positive effects on trabecular bone structure in the context of aging, which occurs despite substantial accumulation of BMAT. These data suggest that the endocrine profile of BMAT, rather than its fatty acid composition, contributes to healthy bone maintenance in aged mice.

## 1 Introduction

With life expectancy rising, age-related diseases are becoming more prevalent (1). Caloric restriction (CR), a nutritional intervention of controlled reduction of calorie intake, has been described to increase lifespan while lowering the risk for cardiometabolic complications and other pathologies (2). A growing area of scientific interest is the identification of CR mimetics, which could deliver the beneficial effects of this intervention without the necessity of reducing food intake (3). However, the impact of CR on bone health remains controversial, as some detrimental effects on bone health have been reported which would limit the applicability of this strategy to improve life expectancy and health-span (2, 4). For instance, consuming a diet low in calories leads to weight loss, which has been associated with reduced bone mineral density (5, 6). Moreover, caloric restriction has paradoxically been associated with increased accumulation of bone marrow adipose tissue (BMAT), which comprises a distinct type of fat cells that accumulates within the bone marrow cavities (7, 8). BMAT displays a characteristic distribution within long bones and has been proposed to exist in distinct subtypes. These observations may contribute to the theory that BMAT may exert detrimental as well as beneficial effects in the bone microenvironment (9–11). The formation of bone marrow adipocytes (BMAds) starts early in childhood in distal parts of the skeleton, forming the so-called constitutive BMAT (cBMAT). This type of BMAT is rich in unsaturated fatty acids and morphologically similar to other depots of white adipose tissue throughout the body, with large unilocular adipocytes (9, 10). As such regions generally contain only adipocytes and few other structures, cBMAT may reflect what is sometimes referred to as ‘yellow bone marrow’. During aging, BMAT accumulation progresses into the proximal regions of the skeleton, leading to the formation of the regulated BMAT (rBMAT). Compared to cBMAT, it features smaller adipocytes and is located in regions with high hematopoiesis, and contains mostly saturated fatty acids (9, 11). Contributing to the complex and controversial role of BMAT in bone health, its expansion is further promoted by pathological factors like obesity and type-2 diabetes mellitus, among others (12, 13). The extent of bone marrow adiposity correlates with lower bone mass, but it remains unclear if BMAT is predictive for fracture risk in obesity and/or diabetes (14, 15).

As discussed above, BMAT paradoxically also increases during CR, and is also found to expand under starvation and in patients with Anorexia nervosa, when other white fat depots are being severely depleted (7, 16, 17). Recently, it has been reported that BMAT also increases during a short-term fasting intervention of 10 days in humans, indicating that BMAT accrual is regulated acutely during energy restriction (18). In such situations, it has been proposed to serve as a local energy source for hematopoiesis and bone turnover when energy supply is limited (10). Moreover, BMAT becomes a significant source of the adipokine adiponectin in calorically restricted animals. Adiponectin is well-known to promote metabolic health, altogether suggesting that BMAT may contribute to CR-induced positive effects via this hormone (7). To investigate the link between BMAT composition, aging and bone quality, we assessed the effects of CR on bone structure, regeneration, and adipocyte accumulation by applying this dietary intervention to young and aged male mice. Our data taken together indicate that BMAT accrual is not necessarily linked to a negative impact on bone health and also suggest that the degree of fatty acid saturation within this fat depot is not a strong prognostic marker of bone health outcomes.

## 2 Materials and methods

### 2.1 Animal housing and tibia osteotomy

All procedures were approved by the ethics committee for animal welfare of the State Office of Environment, Health, and Consumer Protection (State of Brandenburg, Germany). Male C57Bl6J mice were maintained as a local SPF (Specific pathogen-free) colony (original source of animals: Charles River Laboratories, Sulzfeld, Germany) and were housed in a controlled environment (22 ± 2°C, 12/12 h light/dark cycle). Three weeks prior to the caloric restriction, mice were single caged and switched to the NIH-31 rodent open formula diet (Sniff Spezialdiäten GmbH, Soest, Germany, Number: S8022-S030) (19). In order to measure accurate food intake, special baskets were used, that collect food dust at the bottom. After one week of acclimatization, food intake was assessed by weighing the food baskets daily in the morning for two weeks. When starting CR, the diet was switched to the NIH-31/NIA modified diet (Sniff Spezialdiäten GmbH, Soest, Germany, Number: S8022-S032), which is enriched in micronutrients (19). At the start of the CR intervention, young mice were between 8–12 weeks (i.e. 2–3 months) old and aged mice were between 65–78 weeks (i.e. 15–18 months) old. In the first week of CR, food was reduced by 10%, in the second week it was decreased by 20%, and to 40% of the initial food consumption, starting in the third week until the end of the restriction (**Supplementary Figure S1**). CR mice received the weighed-out food just before the beginning of the dark-period. Body weight was monitored weekly. Tibia osteotomies were performed 21 days (i.e. 3 weeks) before the end of the eight-week dietary intervention period. Mice received an analgesic 30 min prior to surgery (buprenorphine; 0.1 mg/kg, i.p.) and the antibiotic clindamycin (100 µl; 1.6 mg/ml, s.c.). Additionally, Tramadol was administered with the drinking water (1 mg/ml) from 2 days before until 3 days after surgery. Anesthesia was induced with an isoflurane/oxygen gas mixture. During the surgery, eyes were covered with ointment and mice were kept on a heating pad at 37°C. The leg area was shaved and disinfected. An incision along the right tibia was made and the bone was exposed using forceps while adjacent muscles were removed by blunt preparation. An internal fixator plate (MouseFix plate 3 hole, RISystem, Landquart, Switzerland) was placed at the proximal part of the tibia, just below the knee ligaments. The fixator was secured by drill-inserting three screws and one pin (MouseFix screw, RISystem, Landquart, Switzerland). The fracture gap was created post-fixation by using a giggly wire saw with a diameter of 0.22 mm which results in a fracture gap of approximately 0.4 mm. The skin was closed using non-resorbable suture material. Mice were placed near an infra-red lamp until the isoflurane anesthesia wore off. For the first three days after the surgery, food pellets were provided in the cage and wounds were controlled daily. At the end of the dietary intervention, mice were killed by cervical dislocation. At the end of the experiment, upon tissue collection, young mice were 16–20 weeks (4–5 months) of age and aged mice were 73–86 weeks (i.e. 17–20 months) of age (**Supplementary Figure S1**). Tissues were isolated, immediately frozen in liquid nitrogen and stored at -80°C until further analysis or placed in a 4% paraformaldehyde solution for 24 hours for subsequent Micro-CT analysis.

### 2.2 Quantitative real-time PCR

For regional tibia gene expression analysis, the tibia was cut into the three regions of interest, using the crest of the tibia to determine the proximal area, and the fibula secession to define the distal area. The remaining tibia shaft was defined as the diaphyseal area. Total RNA was isolated from liquid nitrogen-frozen ground powder of the segments or using an aliquot of 50 mg of powder for whole bone samples. 200 µl Trizol reagent (Thermo Fisher Scientific, Dreieich, Germany) were added, samples were vortexed for one minute, followed by centrifugation for 10 min at 4°C and 12,000 g. Supernatant was transferred into a new tube and 40 µl of chloroform were added for phase separation. Samples were centrifuged for 15 min at 12,000 g and 4°C, followed by RNA precipitation from the collected upper clear phase with 250 μl of isopropanol. RNA was purified by washing in 500 μl of 75% ethanol, dried and solubilized in 30 μl DEPC-treated water. Using a high-capacity cDNA reverse transcription kit (Thermo Fisher Scientific, Dreieich, Germany), RNA was reversely transcribed to cDNA according to the kit instructions. Quantitative real- time PCR was carried out using Maxima SYBR Green/ROX qPCR Master Mix (Thermo Fisher Scientific, Dreieich Germany) on a CFX384 Touch instrument (Bio-Rad, München, Germany). Gene expression was normalized to expression of housekeeping gene TATA box binding protein (Tbp). Primers were designed as intron spanning sequences to specifically amplify cDNA using the sequences listed in **Supplementary Table 1**.

### 2.3 RNA sequencing

For RNA sequencing analysis, DNase digest of purified RNA was performed using the Invitrogen Turbo DNA-free kit (Thermo Fisher Scientific, Dreieich Germany). The Agilent 2100 BioAnalyzer (Agilent RNA 6000 Nano Kit) was used to check for total RNA quality of the samples. Briefly, mRNA was prepared for sequencing using the TruSeq stranded mRNA sample preparation kit (Illumina), with a selected insert size of 120–210 bp, and sequenced on an Illumina HiSeq 4000, as previously described (20). FastQC (version 0.11.8) and MultiQC (V1.6) were used to check the quality metrics of the datasets. Reads containing adapter sequences were filtered out using Trim Galore 0.5.0. Filtered reads were mapped to the mm10 reference genome using the alignment tools STAR (2.6.1). FPKM (Fragments Per Kilobase of transcript per Million mapped reads) values were quantified with StringTie 2.1.3b and count matrix was generated in R (v 4.1.1). For comparison between groups, the FPKM values were normalized by quantile normalization. P-value calculation for differential expression of genes between two groups was performed using Welch’s t-test, with additional Benjamini-Hochberg adjustment using R (v 4.1.1). Differentially expressed genes were selected at a p-value cut-off of < 0.05. Differentially expressed gene lists were entered in the Enrichr web server to identify enriched gene ontology (GO) pathways (Biological processes, cellular compartment and molecular function) (21–23). Identified pathways were sorted by -log10 (adjusted p-Value) and depicted as bar graphs. GO Terms were further analyzed in Revigo to identify connections between pathways and remove redundant terms (24). The color of the bubble corresponds to the -log10 (adjusted p-Value), the size of the bubble represents the LogSize value for the GO Term. The raw data have been uploaded to NCBIs’ Gene Expression Omnibus (GEO) database and can be downloaded with GEO accession number GSE262441.

### 2.4 Fatty acid analysis

Regional fatty acid composition was analyzed using powder-ground samples of the distinct tibia regions described above. 15 mg of powder were homogenized in 200 µl of PBS. 50 µl of the homogenized mixture were transferred into a vial, 1000 µl of distilled water were added, followed by 3000 µl of tert-butyl methyl ether/methanol solution. Samples were mixed for 20 minutes using a rotary shaker (KS 130 basic, IKA Werke GmbH & Co, Staufen, Germany). After centrifugation for 10 minutes at 2,000 g at 15°C, the upper lipid containing layer was transferred in a vial to evaporate the solvent using a sample concentrator (SBH130D/3, Dunn Labortechnik, Asbach, Germany) in a constant stream of N2 at 40°C. The dried lipid pellet was dissolved in 500 µl chloroform and applied to a conditioned column (washing twice with 1000 µl n-hexane and 1000 µl chloroform/2-propanol (2/1, v:v) containing 100 mg aminopropyl-modified silica (Chromabond, MacheryNagel GmbH & Co.,KG, Düren, Germany). Columns were placed on a vacuum elution apparatus (Carl Roth, Karlsruhe, Germany). Neutral lipids and free fatty acids were eluted with four times 1000 µl chloroform/methanol/acetic acid (100/2/2, v:v:v). Solvents were evaporated under a constant stream of N2 and 40°C. For hydrolysis and methylation of fatty acids, the dried samples were dissolved in 200 µl toluene, vortexed and transferred to gas chromatography (GC) vials (IVA Analysetechnik, Meerbusch, Germany). To form fatty acid methyl esters (FAME), 15 µl trimethyl sulfonium hydroxide solution were added. Vials were mixed for 30 minutes at 40°C in a thermomixer (MHR 23, DITABIS, Pforzheim, Germany). Analysis of FAMEs was performed using an Agilent GC Systems 7890A gas chromatograph equipped with 7000 GC/MSC Triple Quad mass spectrometer (Agilent technologies, Waldbronn, Germany) and a flame ionization detector. FAME were separated on a GC capillary column. The fatty acid composition was expressed as area percentage of each FA relative to total area of all detected fatty acids.

### 2.5 Micro-CT analysis

Tibiae were harvested after cervical dislocation and directly fixed in 4% buffered paraformaldehyde/PBS at room temperature overnight. Samples were rinsed with distilled water twice and stored in PBS at 4°C. For osteotomy bones, the fixator plate was then removed. Bone structural parameters were monitored with micro-computed tomography (Micro-CT) sing a SkyScan 1172 instrument (Bruker, Kontich, Belgium). The voxel size was set to 10 µM at a source energy of 80 kV and 130 µA with a beam filtering through a 0.5 mm aluminum filter. Three-dimensional (3D) reconstruction was performed with the software NRecon (Bruker, Kontich, Belgium). Reconstructed images underwent further 2D and 3D analysis using CTAn (CT-Analyser) Software (Bruker, Kontich, Belgium) in specific bone regions. For the analysis of trabecularized bone volumes, the opening of the growth plate in the proximal tibia was identified as starting point to define a consistent VOI. Trabecular regions started 600 µm distal of the growth plate opening and extended for 3500 µm into the tibia. For cortical analysis, the fibula-tibia junction was identified as starting point. The cortical analysis volume extended for 1000 µm toward the distal end. For osteotomy analyses, the opening of the cortex was identified on both ends of the osteotomy gap, which was used to identify the size of the callus region. Either the whole fracture gap, defined as the volume of woven bone within the callus volume, was analyzed or a central region of 250 µm or 100 µm around the callus mid-line was used.

### 2.6 Osmium tetroxide staining

Osmium tetroxide staining was carried out according to a published protocol (25). After Micro-CT analysis of mineralized bone, tibiae were decalcified using a 14% EDTA solution at 4°C for 14 days, changing the solution every 3–4 days. Decalcified tibiae were washed for 30 minutes with cool tap water. Next, tibiae were transferred into a falcon containing 1200 µl Sorensen’s Phosphate Buffer (Science Services, München, Germany). 400 µl of a 4% Osmium tetroxide solution, which resulted in a 1% Osmium tetroxide solution, was added for 48 hours. Bones were transferred into a new tube containing 2000 µl Sorensen’s Phosphate Buffer for 3 hours. This step was repeated twice and Micro-CT measurement followed. After reconstruction of the Micro-CT data, three volumes of interest were selected, dividing the tibia into three regions as described above. BMAT volumes for each area were quantified and the ratio of BMAT volume over marrow volume (tissue volume – bone volume) was calculated.

### 2.7 Statistical analyses

Statistical analysis was performed using the GraphPad Prism (versions 9 and 10) software. Data are typically presented as means ± standard error of the mean (SEM), unless stated otherwise. Comparisons between multiple groups were carried out using a 2-way or a 3-way ANOVA with Tukey’s multiple comparisons testing. Thus, different tests of significant variation are considered in the results: ANOVA initially tests for interaction between the individual factors within the test (e.g. typically diet and age in our analysis), where a significant p-value refers to a significant interaction between both factors. Secondly, group effects of the two (or three) independent variables are evaluated by ANOVA to determine whether a factor in itself may explain some of the variance in the results. Significant p-values for the individual variables indicate that the factor is a significant driver of variation independent of other factors within the experiment.

## 3 Results

### 3.1 Caloric restriction leads to increased adipocyte accumulation in the proximal tibia

**Figure 1:**
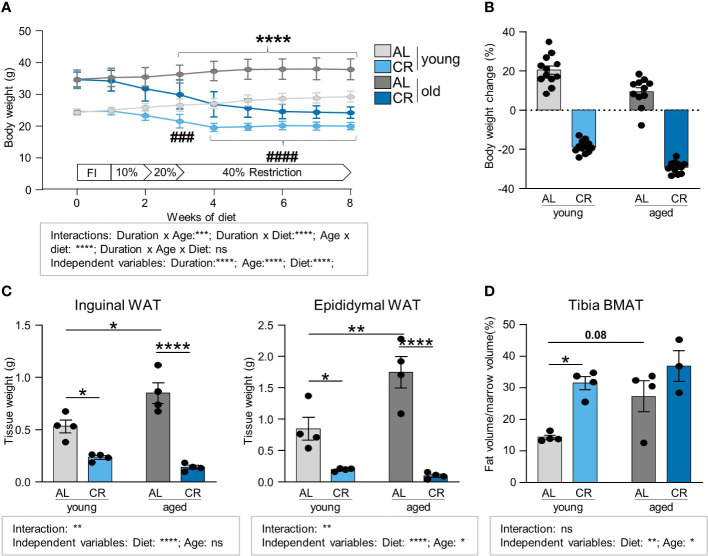
Caloric restriction leads to the loss of white adipose tissue depots while increasing tibia BMAT. **(A)** Body weight development of young (starting age: 8–12 weeks) and aged (starting age: 15–18 months) mice during eight weeks of caloric restriction (CR) compared to *ad libitum* fed (AL) age-matched control mice. Light gray lines/bars indicate young AL, light blue indicate young CR. Dark gray/blue lines/bars indicate CR mice in corresponding aged groups (applies to all Figure panels; AL aged: n=15; all other groups: n=16). Data are depicted as mean ± SEM. ****P<0.001 indicates significant differences between old AL vs. CR; ^###^P<0.001; ^####^P<0.0001 indicates significant differences between young AL vs. CR by multiple comparisons testing after 3-Way ANOVA. **(B)** Percent body weight change after eight weeks of diet feeding of CR and AL diets compared to starting weight (AL aged: n=15; all other groups: n=16). **(C)** Inguinal (left panel) and epididymal (right panel) white fat pad weights after 8 weeks of CR (n=4). **(D)** Micro-CT analyses of tibial BMAT volumes normalized to marrow volume (CR aged: n=3; all other groups: n=4). Data are depicted as mean ± SEM. *P<0.05; **P<0.01; ***P<0.001; ****P<0.0001 as indicated (2-way ANOVA). Source of variation analysis summarized in grey boxes under each panel for significances of factor interaction and for the independent variables by 2-way or 3-way ANOVA (diet, age, duration of diet; ns, not significant; *P<0.05; **P<0.01; ***P<0.001; ****P<0.0001).

To test a dietary application that promotes marrow adipogenesis and at the same time improves systemic metabolic health, CR was applied to cohorts of young and old mice, aged 8–12 weeks or 15–18 months, at the start of the intervention, respectively (**Supplementary Figure S1**). During CR, young and old mice displayed the expected and significant decrease in body weight approximately between weeks 3 and 4, and stabilized after that until the end of the dietary intervention (**Figure 1A**). Statistical three-factor analysis of variation showed significant interactions between all pairs of the three factors diet, age and duration of diet. Therefore, only pairwise comparisons after multiple testing are considered as it is difficult to evaluate the significance of contributions of individual factors. These direct comparisons show significantly reduced body weights in CR groups of both ages starting in week 3. Throughout the full eight weeks of CR, young mice lost on average of 19% of initial body weight, while aged mice lost approximately 27%. Young mice fed *ad libitum* gained 20% of body weight, and old mice fed *ad libitum* gained around 5% of body weight during the eight weeks (**Figure 1B**). Loss of fat mass was verified in inguinal and epididymal white fat depot weights upon CR, but two-factor analysis showed significant interactions between the two independent factors diet and age: Aged *ad libitum* fed mice had significantly greater epididymal and inguinal fat mass, while CR significantly reduced the white fat depots in both young and aged mice (**Figure 1C**). Diet effects were highly significant as independent variable as well in multiple comparison testing, whereas the factor age produced fewer significances which were only found in *ad libitum* fed old mice compared to young mice. Conversely, BMAT in the tibia was mildly increased upon aging, but did not reach statistical significance in this multi-group comparison, whereas the increase of tibial BMAT upon CR was more pronounced, leading to a significant increase in young animals that approximated the overall level of BMAT found in aged mice under CR (**Figure 1D**). Tibia BMAT displayed no interaction between CR and aging. More importantly, unlike white fat, both factors showed the same direction of regulation and both factors, age and diet, showed significantly increased BMAT formation as independent variables.

### 3.2 Caloric Restriction diminishes trabecular bone loss in aged mice

**Figure 2:**
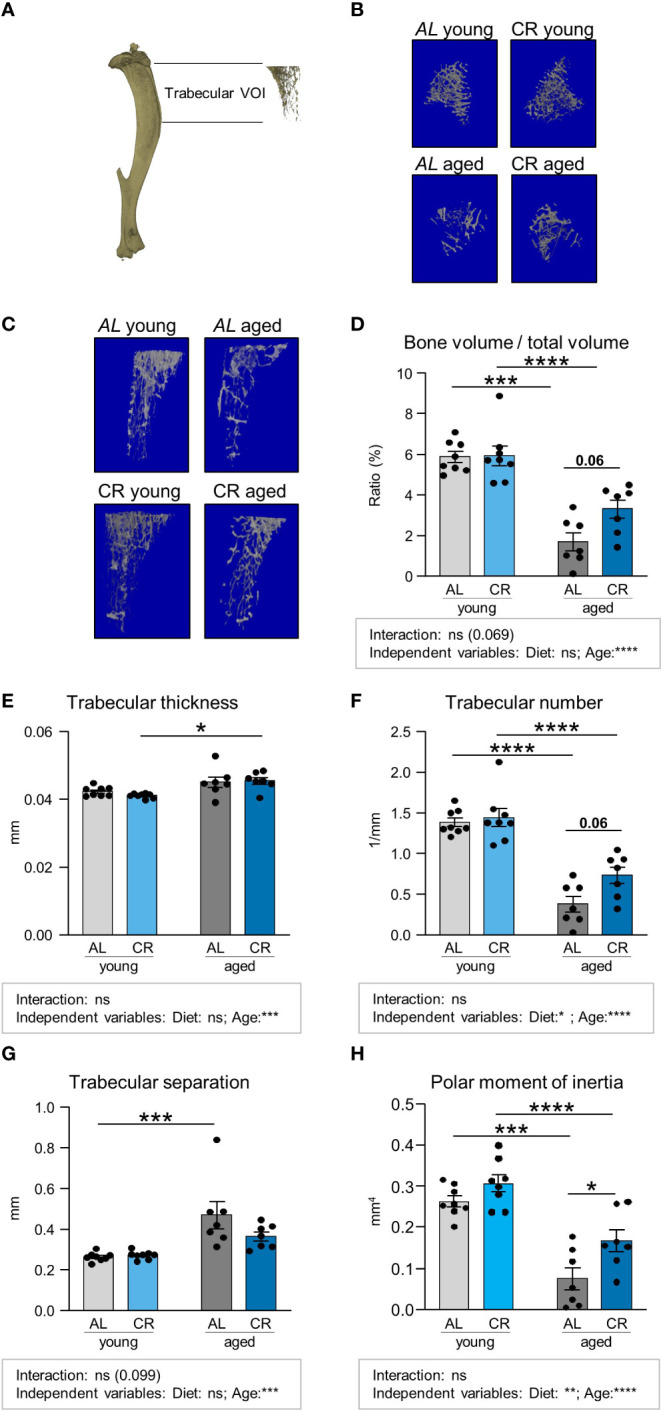
Caloric restriction prevents age-associated trabecular bone loss. **(A)** Depiction of Micro-CT analysis of volume of interest (VOI) including trabecularized regions of the metaphyseal and diaphyseal proximal tibia, excluding the cortical portion of the VOI. **(B, C)** representative 3D-renders of the analyzed trabecular regions. **(D)** Quantifications of bone volume normalized to total volume area. Light gray bars indicate young *ad libitum* fed (AL) controls (n=8), light blue bars indicate young CR (n=8), dark gray bars indicate aged AL mice (n=7), and dark blue bars indicate aged CR mice (n=7; applies to all Figure panels). **(E)** Quantification of trabecular thickness, **(F)** trabecular number, **(G)** trabecular separation, and **(H)** polar moment of inertia in the indicated VOI. Data are depicted as mean ± SEM. *P<0.05; **P<0.01; ***P<0.001; ****P<0.0001 indicate significant differences by multiple comparisons testing after 2-way ANOVA. Source of variation analysis summarized in grey boxes under each panel for significances of factor interaction and for the independent variables (diet and age; ns, not significant; *P<0.05; **P<0.01; ***P<0.001; ****P<0.0001).

**Figure 3:**
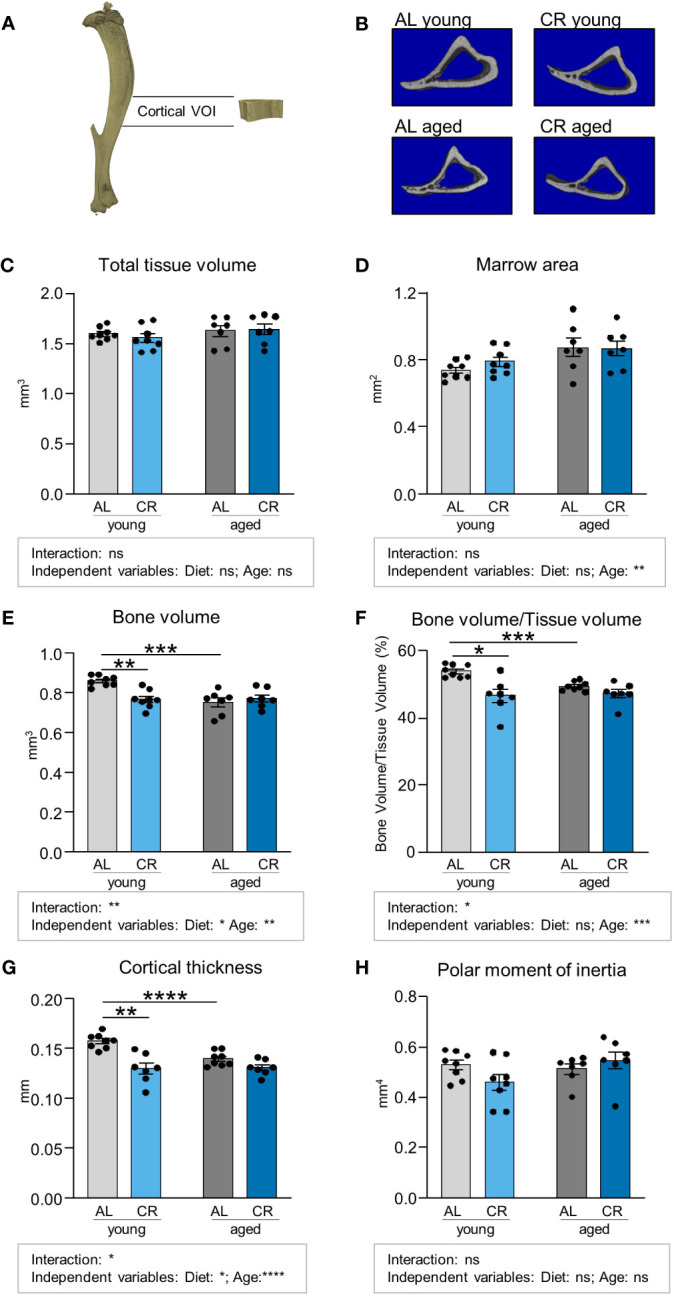
Caloric restriction reduces cortical bone in young mice. **(A)** Depiction of Micro-CT analysis of volume of interest (VOI) of cortical bone in the mid-shaft, diaphyseal region of the tibia. **(B)** Representative cross-sectional 3D-rendered images of the analyzed cortical bone region. **(C)** Quantifications of total analyzed bone tissue volume. Light gray bars indicate young *ad libitum* fed (AL) controls (n=8), light blue bars indicate young CR (n=8), dark gray bars indicate aged AL mice (n=7), and dark blue bars indicate aged CR mice (n=7; applies to all Figure panels). **(D)** Quantification of non-mineralized bone marrow volume, **(E)** mineralized bone volume, **(F)** Quantification of bone volume/tissue volume. **(G)** cortical thickness, and **(H)** polar moment of inertia within indicated VOI. Data are depicted as mean ± SEM. *P<0.05; **P<0.01; ***P<0.001; ****P<0.0001 indicate significant differences by multiple comparisons testing after 2-way ANOVA. Source of variation analysis summarized in grey boxes under each panel for significances of factor interaction and for the independent variables (diet and age; ns, not significant; *P<0.05; **P<0.01; ***P<0.001; ****P<0.0001).

The tibia harbors constitutive and regulated BMAT in specific regions (9). Using micro-computed tomography (Micro-CT), the tibia bone microstructure was analyzed by assessing two distinct regions, a proximal region encompassing the metaphyseal to diaphyseal tibia, which is trabecularized throughout the analyzed volume (**Figures 2A-C**; **Supplementary Figure S2**, **Supplementary Figure S3**), and a more distal region within the diaphyseal shaft, which mainly contains cortical bone (**Figure 3**; **Supplementary Figure S4**). Trabecular bone volume was significantly decreased in aged mice on both diets, confirmed by significant reductions for age, but not diet, as independent variable, which is consistent with the literature (26, 27). While not reaching statistical significance, there was a trend (p=0.06) for partial amelioration by CR in the aged tibia, while CR had no effect on this parameter in young mice (**Figure 2D**). Trabecular thickness was increased in aged mice in direct multiple testing, i.e. regardless of CR, and was significantly increased as independent variable that did not statistically interact with the diet factor (**Figure 2E**). This effect is again consistent with the literature (28). The overall trabecular bone loss during aging was also reflected by significantly decreased trabecular numbers, increased trabecular separation and reduced polar moment of inertia, which describes the resistance of the bone to withstand torsional deformation as calculated from micro-CT results (**Figures 2F-H**). There was no significant interaction between the factors diet and age for these parameters. Age-effects were significant in multiple comparisons testing in both diets and for age as independent variable. The effect of diet showed a non-significant trend to be increased (p = 0.06) for trabecular number and was significantly increased for polar moment of inertia in aged, but not young, mice after CR, altogether suggesting improvement of bone quality in aged tibia bones of male mice upon CR for these parameters. This was further confirmed by the significant effect of diet as independent variable for these two parameters. The beneficial effect of CR on aged trabecular bone was also reflected in the polar moment of inertia. While generally reduced upon aging, aged mice undergoing CR showed a significant increase in this parameter compared to the aged *ad libitum* fed group (**Figure 2H**). In summary, the detrimental effect of aging on trabecular bone was pronounced in all analyzed parameters, as indicated by the significance levels. CR had displayed no significant effects in young mice while displaying some trends or significances for improvement in aged mice, depending on the parameter measured, which was further confirmed when analyzing the significances of diet as independent variable.

We subsequently conduced micro-CT analyses of the cortical tibia bone volume, which showed no clear effects of diet or age upon initial inspection of total tissue volumes (**Figures 3A-C**; **Supplementary Figure S4**). However, a detailed analysis of marrow area showed that age, as diet-independent factor, resulted in a significant increase of this parameter (**Figure 3D**). The three parameters cortical bone volume, ratio of cortical bone volume over total tissue volume, and cortical thickness were significantly decreased by aging in *ad libitum* fed mice and also in young mice when comparing AL and CR, while CR had no impact on these parameters when administered to aged mice (**Figures 3E-G**). Interactions between factors age and diet were significant for these parameters. Age as independent factor of variation was significant for all three parameters, while the variation explained due to diet was significant only for cortical bone volume and cortical thickness. Altogether these results suggest that diet and age act as at least partially independent negative regulators of cortical parameters in comparison to young mice with *ad libitum* feeding. The polar moment of inertia of this region, as an indicator for torsional bone strength, was not altered by aging and CR, suggesting that the ability of the cortical bone to withstand torsional loads remained intact (**Figure 3H**).

### 3.3 Caloric Restriction increases adipogenic and mitochondrial gene expression in CR mice

**Figure 4:**
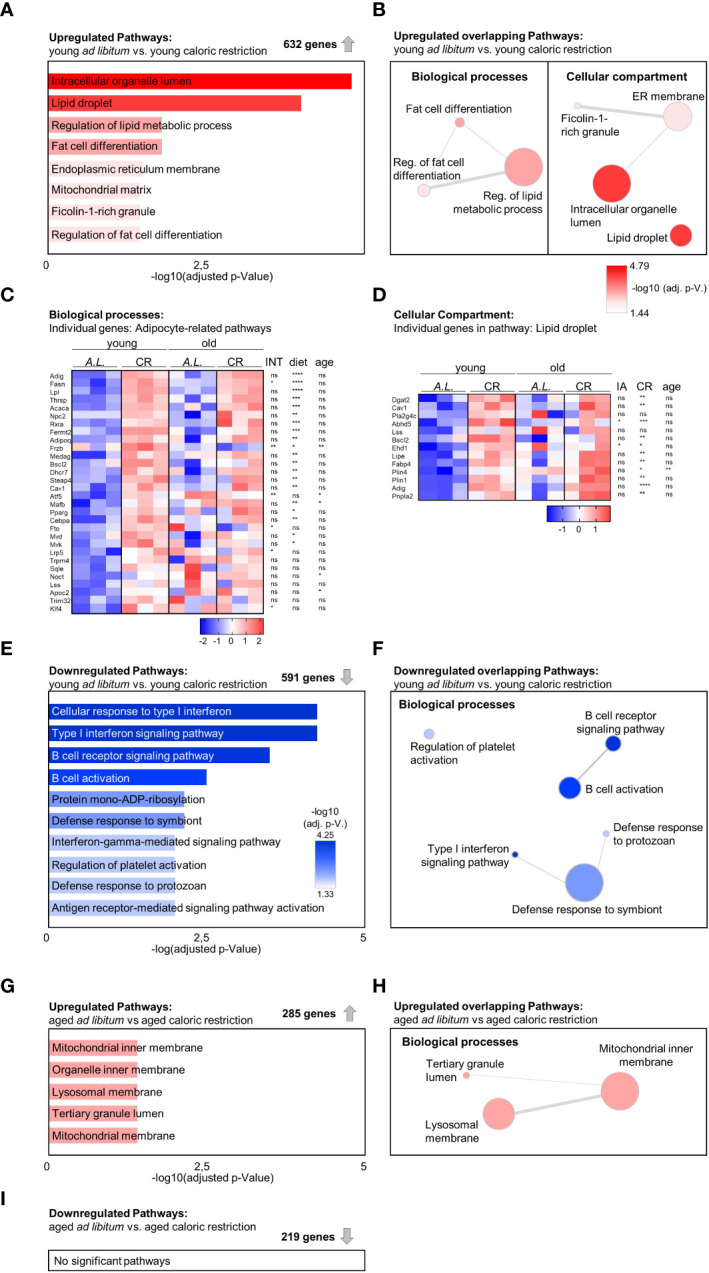
Increased adipogenic gene expression signature in tibia due to aging and caloric restriction. **(A)** Gene Ontology (GO) term enrichment analysis within 632 significantly upregulated genes when comparing young mice receiving CR to the young *ad libitum* fed (AL) mice (n=3). A summary of all significantly enriched GO terms from the domains ‘Cellular component’ and ‘Biological process’ is shown (bar length and color of the bar indicates adjusted p-value). **(B)** Identification of GO term similarity analysis within the two GO domains using Revigo [as shown in **(A)**]. Bubble color indicates -log10 of adjusted P-value for each identified GO term; bubble size indicates the frequency of the GO term in the underlying GO annotation (GOA) database; line width indicates the degree of similarity between terms. **(C)** Heatmap visualization of all four intervention groups summarizing all expressed genes related to adipocytes and lipid metabolism from GO analysis of enriched terms within the ‘Biological process’ domain [as shown in **(B)** (left); data reported as z-scores]. Source of variation analysis summarized on right next to heat maps for significances of factor interaction (INT) and for significances of the effects of independent variables diet and age (ns = not significant; *P<0.05; **P<0.01; ***P<0.001; ****P<0.0001; also applies to subsequent panel **D**). **(D)** Heatmap visualization of all four intervention groups summarizing all expressed genes related to the term ‘Lipid droplet’ from GO analysis of enriched terms within the ‘Cellular component’ domain [as shown in **(B)** (right); data reported as z-scores]. **(E)** GO term enrichment analysis within 591 significantly downregulated genes when comparing young mice receiving CR to the young *ad libitum* fed (AL) mice. A summary of the top 10 significantly enriched GO terms from the domains ‘Cellular component’ and ‘Biological process’ is shown (bar length and color of the bar indicates adjusted p-value). **(F)** Identification of GO term similarity analysis within the GO domains ‘Biological process’ using Revigo [using terms as shown in **(E)**]. Bubble color indicates -log10 of adjusted P-value for each identified GO term; bubble size indicates the frequency of the GO term in the underlying GOA database; line width indicates the degree of similarity between terms. **(G)** GO term enrichment analysis within 285 significantly upregulated genes comparing aged *ad libitum* fed to aged CR mice (n=3). Significantly enriched terms only occurred within the GO domain ‘Biological process’ (bar length and color of the bar indicates adjusted p-value). **(H)** Identification of GO term similarity analysis within the GO domain ‘Biological process’ using Revigo [as shown in **(E)**]. Bubble color indicates -log10 of adjusted P-value for each identified GO term; bubble size indicates the frequency of the GO term in the underlying GOA database; line width indicates the degree of similarity between terms. **(I)** A similar gene set analysis was conducted for the 219 significantly downregulated genes in aged CR mice compared to the aged *ad libitum* (AL) fed group, but yielded no significantly enriched GO pathways (n=3).

To investigate molecular changes in bone tissue following CR, a transcriptomic analysis of tibiae from the four conditions was performed. First, the effects of CR in young animals were analyzed by comparing the two groups of young mice, i.e. mice with *ad libitum* food access to CR mice. Gene ontology (GO) analysis of the 632 significantly upregulated genes led to eight enriched gene sets, four of which were related to adipocytes or lipid metabolism (**Figure 4A**). Subsequent interaction analysis of overlapping genes between these adipose-related gene sets confirmed these results, altogether reflecting the notion that CR leads to robust induction of bone marrow adipogenesis (**Figures 4B-D**). When comparing the expression changes of the genes resulting from this network analysis in both age groups, a similar pattern of increased adipogenesis-related gene expression emerged in the aged animals under CR compared to the age-matched control mice with *ad libitum* food access, albeit less pronounced compared to young mice, as aging itself is a potent inducer of adipogenesis in the bone (**Figures 4C, D**). Of note, only very few genes showed statically significant interactions between diet and age, whereas most genes showed significant upregulation when considering diet (i.e. CR compared to AL) as independent variable, while variation partially explained due to the factor age was only significant in a small number of genes. Specifically, 20 of the 30 genes summarized in the “adipocyte-related pathways” were significantly elevated through CR, while aging only induced expression of 5 genes (**Figure 4C**). Similar results were obtained in the pathway “lipid droplet”, where CR significantly induced 11 of the 13 genes while aging induced only one gene (**Figure 4D**). CR led to a significant downregulation of 591 genes in young mice with *ad libitum* food access compared to young CR mice, and gene set functional scoring showed these genes to be involved in B-cell function and interferon type 1 signaling (**Figure 4E**). The combination of redundant pathways, i.e. those that relied on largely overlapping gene sets, from this analysis showed that five of the six remaining gene sets were related to immune system functions, illustrating the potential immune-suppressant effects of CR (**Figure 4F**).

To determine which pathways were affected by CR in aged mice, a similar pathway analysis was carried out comparing transcriptomic data from aged animals on control diet to animals on CR, leading to 285 significantly upregulated genes and five significantly enriched pathways, two of which were related to mitochondrial function (**Figure 4G**). After filtering of redundant gene sets and genes, the three remaining biological processes were linked to the inner mitochondrial matrix, altogether suggesting that CR induces expression of genes linked to mitochondrial function in bones of aged mice, which could reflect improved metabolic functions in the aged bones of mice on CR (**Figure 4H**). A similar analysis of the 219 significantly downregulated genes resulted in no significant pathway changes (**Figure 4I**).

### 3.4 Caloric restriction enhances unsaturated fat accumulation in the proximal tibia

**Figure 5:**
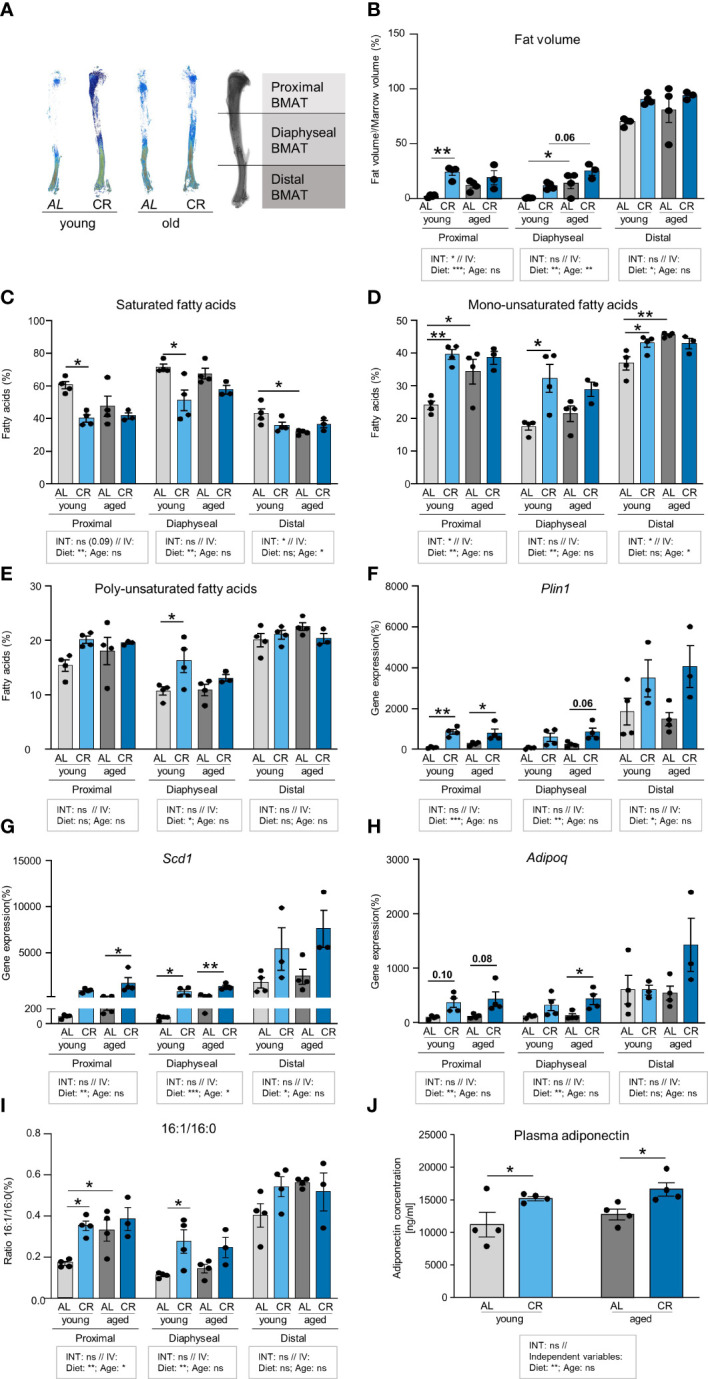
Caloric restriction and aging enhance fatty acid desaturation in tibial neutral lipids. **(A)** Representative densitometric images of Micro-CT analyses of tibial BMAT using Osmium tetroxide staining. Tibiae were subdivided into three regions representing the proximal, diaphyseal, and distal BMAT volumes. **(B)** Quantification of BMAT accumulation in the different tibia regions as Osmium tetroxide stained volume normalized to marrow volume. Tissue volume was quantified by Micro-CT scanning. Tibiae were subsequently decalcified and stained with Osmium tetroxide for BMAT quantification. In the indicated tibial regions, light gray bars indicate young *ad libitum* fed (AL) controls (n=4), light blue bars indicate young CR (n=4), dark gray bars indicate aged AL mice (n=4), and dark blue bars indicate aged CR mice (n=3; applies to all Figure panels). **(C)** Region-specific quantification of all saturated fatty acids (SFA) in the tibia bone of mice after the four interventions (individual data summarized in **Supplementary Table 2**). **(D)** Region-specific quantification of all mono-unsaturated fatty acids (MUFA) in the tibia bone of mice after the four interventions (aged CR: n=3; all other groups: n=4; individual data summarized in **Supplementary Table 2**). **(E)** Region-specific quantification of all poly-unsaturated fatty acids (PUFA) in the tibia bone of mice after the four interventions (individual data summarized in **Supplementary Table 2**). **(F)** mRNA expression of lipid droplet surface marker *Plin1*. **(G)** mRNA expression of fatty acid desaturation enzyme *Scd1*. **(H)** mRNA expression of adipokine *Adipoq.* **(I)** Ratio of palmitoleic over palmitic acid (16:1/16:0) from analysis shown in panels **(C, D)**. **(J)** Plasma concentrations of adiponectin in young and aged mice with CR or without (i.e. AL), assessed by ELISA (n=4). Data are depicted as mean ± SEM (mRNA expression analysis: proximal region: aged CR: n=3–4; all other groups: n=4; diaphyseal region: n=4; distal region: aged CR and young CR: n=3; all other groups: n=4; 16:1/16:0 ratio: CR aged: n=3; all other groups: n=4). *P<0.05; **P<0.01 indicate significant differences by multiple comparisons testing after 2-way ANOVA. Source of variation analysis summarized in grey boxes under each panel for significances of factor interaction (INT) and for the independent variables (IV, i.e. diet and age; ns, not significant; *P<0.05; **P<0.01; ***P<0.001; ****P<0.0001).

As BMAT features regionally distinct degrees of fatty acid saturation within storage lipids (29), tibia bones were subdivided into three region segments to examine the impact of CR and aging on marrow adipogenesis and the regionally distinct fatty acid composition of this adipose depot more closely (**Figure 5A**). Multiple comparison testing showed few significant differences: CR increased fat accumulation in the proximal tibia of young mice, which does not normally feature high levels of BMAT. In the diaphyseal region, aging elevated BMAT accumulation in *ad libitum* fed mice. The distal tibia showed no significant differences between the groups (**Figure 5B**). A more detailed statistical analysis showed that diet and age interacted in the proximal tibia, but only diet as independent variable explained a significant proportion of variance. No factor-interaction occurred in diaphyseal and distal segments. Again, diet as independent variable explained a significant increase in BMAT volume in both regions, whereas age also explained a significant increase in BMAT volume in the diaphyseal region. Together, these results indicate that the contribution of CR as the dietary intervention explained a higher degree of BMAT accumulation compared to age alone (**Figure 5B**).

An analysis of the lipid composition in all three regions showed lower levels of saturated fatty acids (SFA) in the proximal and diaphyseal region of young CR mice, which was accompanied by a significant increase of mono-unsaturated fatty acids (MUFA) (**Figures 5C, D**). MUFA were also significantly increased in aged compared to young AL mice in proximal and diaphyseal regions. Comparing these two factors, analysis of variance showed only very few interactions between the two variables, age and diet. More interestingly, CR as independent variable consistently explained significant differences in SFA and MUFA compared to AL mice in proximal and diaphyseal tibia, which contains mainly rBMAT, Conversely, age as independent variable explained significant differences in SFA and MUFA in the distal tibia, which contains mainly cBMAT. Poly-unsaturated fatty acids (PUFA) showed very few significant differences, and were only increased in the diaphyseal region of young mice after CR compared to *ad libitum* food access (**Figure 5E**). Taken together, CR and aging were both associated with a higher level of fatty acid desaturation, but in distinct regions of the tibia.

To further evaluate this effect, gene expression analysis of the corresponding tibia regions revealed a pattern consistent with BMAT abundance, with high mRNA levels of adipocyte marker genes perilipin-1 (*Plin1*), stearoyl-CoA-desaturase (*Scd1*) and adiponectin (*Adipoq*) in the distal end of young control tibia samples (**Figures 5F-H**). Mirroring the impact of CR on BMAT accumulation, expression of *Plin1* was significantly induced by CR in young and aged mice in the proximal tibia. When testing the impact of the dietary intervention as independent variable across both age groups, CR induced *Plin1* expression significantly, with the strongest effect in the proximal tibia, while aging as independent variable had no significant effect (**Figure 5F**). Expression of the desaturase enzyme gene, *Scd1*, was significantly increased in the diaphyseal tibia of young and old mice when receiving CR, and a similar significant trend was observed in old mice on CR in the proximal tibia (**Figure 5G**), which supports the observation of a general loss of SFAs in CR-exposed tibiae. Again, when testing the impact of CR as common factor, independent of aging, elevated *Scd1* expression occurred in all regions of the tibia. The effect was most pronounced in the diaphyseal region, which was also the only region where the factor age explained some of the variation between groups (**Figure 5G**). While we did not assess SCD1 protein directly, the ratio of palmitoleic over palmitic acid (16:1/16:0) has been used as an indirect measure of SCD1 activity (30). In the proximal tibia, diet and age as independent variables both significantly increased the ratio, suggesting that both were jointly driving increased desaturation. In the diaphyseal region, only diet as independent variable remained significant to explain the increased ratio, whereas no effects occurred in the distal tibia (**Figure 5I**). Expression of *Adipoq* mRNA, which encodes for the adipokine adiponectin, was only mildly, but not significantly elevated in the proximal region of both age groups and was only significantly increased in the diaphyseal region of aged mice on CR compared to *ad libitum* feeding. When testing diet and age as independent variables, diet, but not age, significantly increased *Adipoq* in the proximal and diaphyseal tibia, whereas no effect was found for age (**Figure 5I**). The absence of an effect of age alone could suggest that the expression of this adipokine, which has previously been linked to CR and BMAT (7), could mediate some of the beneficial effects of CR in bone. Indeed, when we measured plasma adiponectin concentrations in these mice, we found levels to be consistently increased in mice on CR in both age groups, further promoting the notion that CR could create a healthier endocrine profile in BMAT (**Figure 5J**). In summary, while both aging and CR stimulated the expression of general adipocyte marker genes in some regions of the tibia, CR exerted a more pronounced induction compared to aging alone. Elevated expression of the desaturase enzyme *Scd1* in tibia regions of aged mice and after CR could help explain the increase in unsaturated fatty acids. Conversely, CR, but not age, induced expression and secretion of adiponectin.

### 3.5 Caloric Restriction does not impair tibia fracture healing

**Figure 6:**
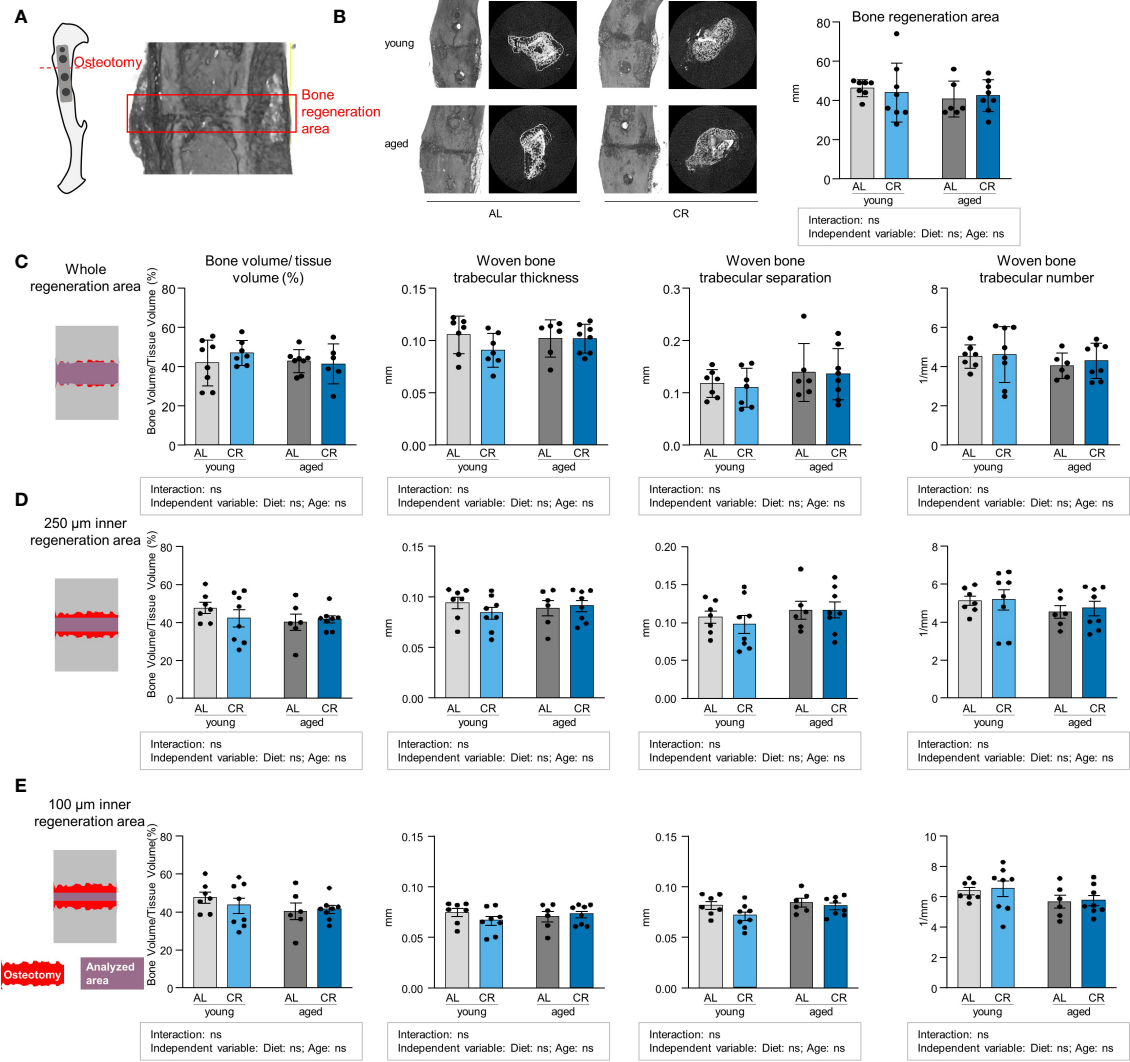
Non-critical bone defect healing is not affected by caloric restriction or aging. **(A)** Internal fixator plate-stabilized tibia osteotomy model and Micro-CT analysis of representative fracture gap 21 days post-osteotomy. **(B)** Representative images of the osteotomy area from all four intervention groups and quantification of fracture gap width as measured by assessing the breakdown of regular cortical bone on both fracture ends. Light gray bars indicate young *ad libitum* fed (AL) controls (n=7), light blue bars indicate young CR (n=8), dark gray bars indicate aged AL mice (n=6), and dark blue bars indicate aged CR mice (n=8; applies to all Figure panels). **(C)** Micro-CT analysis of tissue-volume normalized bone volume, trabecular thickness, separation and number in entire osteotomy/woven bone volume. **(D)** Micro-CT analysis of tissue-volume normalized bone volume, trabecular thickness, separation and number in osteotomy/woven bone volume corresponding to a volume of 250 µM surrounding the mid-line of the callus volume. **(E)** Micro-CT analysis of tissue-volume normalized bone volume, trabecular thickness, separation and number in osteotomy/woven bone volume corresponding to a volume of 100 µM surrounding the mid-line of the callus volume. Data are depicted as mean ± SEM (statistical testing: 2-way ANOVA). Source of variation analysis summarized in grey boxes under each panel for significances of factor interaction and for the independent variables (diet and age; ns, not significant).

Aging and CR have independently been linked to impaired fracture healing, and increased BMAT has also been correlated with adverse fracture healing outcomes (31–33). However, such analyses frequently assess bone regeneration in critical-size defects where non-union is expected. In order to relate the study results to an anatomically correctly repositioned bone healing scenario that corresponds to the clinical gold standard of fracture treatment, an internal plate-stabilized osteotomy with a fixed, relatively narrow 400 µm gap was chosen for this study (**Figure 6A**). Accordingly, the width of the fracture gap showed no significant differences between the four groups (**Figure 6B**). However, Micro-CT analysis revealed no differences in bone volume/tissue volume ratios and the parameters woven bone trabecular thickness, -separation and -number when assessing an area covering the entire fracture region (**Figure 6C**). We subsequently assessed the microstructure of the fracture callus using analysis modes that included smaller areas of the regions surrounding the fracture gap to determine whether specific areas of the osteotomy callus were affected differently, but similar results were obtained when the analysis was carried out in more central regions of the fracture (**Figures 6D, E**). Overall, these data show that bone healing is fully intact within this tibia osteotomy model, indicating that endogenous repair mechanisms in a non-critical bone defect remain unaffected despite an accumulation of BMAT induced by either CR or aging.

## 4 Discussion

The effects of caloric restriction, a life span-extending dietary intervention, on bone health remain only partially understood. We here assess the impact of this intervention on bone marrow adipogenesis, bone structure, function and fracture healing in young and aged mice. We show that a relatively short-term CR compares to or even surpasses the age-related extent of marrow adipogenesis in specific tibia bone regions, even in young mice, but produces biochemical and molecular characteristics in BMAT partially overlapping with those observed in aged bones. CR exerts regionally and age-distinct effects in the tibia, which is highly prone to marrow adipogenesis. Cortical bone is negatively affected by CR in young mice, leading to a reduced bone area and cortical thickness, but CR does not further impair this bone compartment in aged mice. Conversely, CR reduces age-related trabecular bone loss, reflected in a higher trabeculae number and increased resistance to deformation.

In our study, we demonstrate that CR affects the trabecular and cortical bone differentially in the context of aging. This could be partially due to ongoing skeletal maturation in C57Bl6 mice, which is only fully completed by 12 months of age, when biomechanical parameters peak before starting an age-dependent to decline (28). Tibia length, mineral mass and cortical cross-sectional area are described to reach their highest value at 6 months of age in mice. The trabecular bone seems to reach maturity earlier, peaking at around 6–8 weeks of age (28, 34). As young mice under CR displayed reduced cortical, but not trabecular, bone, our observation supports the conclusion that nutrient withdrawal prior to reaching peak bone mass and maturity exerts detrimental effects by inhibiting complete bone maturation. CR could, however, exert beneficial effects on fully mature bone areas, thus mirroring the reports of beneficial effects of this intervention on age-related pathology in many other tissues. For instance, this would explain cortical bone loss upon CR in young mice while no effect is evident in CR on cortical parameters in bones of aged mice where the cortex was fully mature. Trabecular bone loss is spared upon CR in young mice as peak trabecular mass has already been reached before the start of the restriction intervention. Previous studies reported similar observations for CR in young adult mice, which led to a reduction in the cortical thickness of the femur and the spine, whereas trabecular areas were protected (35). In the same line, 3-week-old mice under CR showed signs of growth retardation and reduced trabecular and cortical bone mass (36). Moreover, the negative effects of CR on cortical bone might diminish over time. While four weeks of CR acutely reduced cortical bone mass, the effects were blunted when CR was prolonged for 20 or even 74 weeks, when trabecular number and thickness increased significantly (37). Although the duration of CR is different, this is to some extent in accordance with our findings in aged mice. In a life-long CR intervention study, it is conceivable that CR reduced bone mass in the first 12 months of life, potentially by decreasing the bone formation rate. When CR was applied until death, mice showed a higher bone mass, suggesting a decreased bone turnover rate during aging, thereby protecting the mineralized bone (38).

The osteotomy technique used in our study, which applied an internal fixator plate and a bone defect width of approximately 400 μm, represents an anatomically correctly repositioned fracture with stable fixation, altogether mimicking the gold standard of clinical fracture care. Under such conditions, fracture healing progression was normal in all groups, even in aged mice where the non-fractured tibia shows clear deterioration of trabecular and cortical areas in the *ad libitum* fed groups, i.e. independent of CR, suggesting that the regenerative capacity of bones *per se* remains intact. In a femur fracture model using a wider fracture gap of 700 µm in 24 months old mice, aging showed a robust negative effect on fracture healing, which might be explained by the larger fracture gap, older animals used in the study or a combination of both (39). Thus, our data show that tibia bone regeneration of a repositioned osteotomy is not negatively affected by CR or, unexpectedly, by aging, indicating that successful bone regeneration can still be achieved even in the presence of several detrimental factors. As a potential inhibitory factor of bone healing, we observed that CR increased BMAT accumulation in the tibia and was an even more potent inducer of marrow adipogenesis than aging. Several studies have reported the BMAT-inducing effect of CR in different species (7, 36). Besides the increase in total tibia fat content, adipocyte differentiation markers and lipid droplet markers were markedly upregulated. An induction of adipogenesis genes was also shown in the transcriptomic analysis of tibia of mice undergoing 30% CR for 12 weeks. In contrast to our analysis, the study also reports an induction of genes related to the skeletal system, which might be explained by the milder restriction (40). In our data, the increase in BMAT formation upon CR was strongest in the proximal regions of the tibia, corresponding to the area where the osteotomy was introduced, which has also been observed previously (17). Importantly, we found an increase in monounsaturated fatty acids upon CR and a corresponding decrease in saturated fatty acids in the proximal and diaphyseal region of the tibia, which mainly harbors regulated BMAT. rBMAT, which also expands in the proximal tibia during aging and obesity, has been described to contain more saturated fatty acids (9). The fatty acid composition in the proximal part of the tibia of CR mice was therefore more similar to distal BMAT, which indicates that the type of fat that forms upon CR might be more related to cBMAT, which arises early in life and is thought to play a more physiological beneficial role compared to rBMAT (17, 41). Our data further show that fatty acid desaturation might occur directly in the bone as the expression of stearoyl-Coa-desaturase 1 (*Scd1*) is upregulated upon CR and the ratio of monounsaturated palmitoleic acid over the saturated palmitic acid is consistently elevated. Further evidence for a potentially protective role of BMAT during CR was shown in a recent study using a mouse model of BMAT-specific deletion of the gene encoding for adipocyte triglyceride lipase (*Atgl*) which is a key enzyme of lipid mobilization in adipocytes. These mice displayed high bone marrow adiposity, but when challenged with CR they experienced trabecular bone loss, which was ascribed to impaired lipolysis and provision of fatty acids as a substrate (40).

Notably, some of the biochemical changes observed in BMAT following CR are similar to those seen in aged tibia BMAT, however the effects of CR were generally more pronounced than those of aging. Additionally, we observed a shift toward increased levels of unsaturated fatty acids and a decrease in saturated fatty acids, which would somewhat argue against the conclusion that the pattern of lipid saturation was a key driver of improved bone quality in aged mice on CR. Another important function of BMAT could be the secretion of adipokines like adiponectin. Adiponectin is known for its cardio-metabolic benefits and is especially high in individuals with low levels of peripheral fat depots (42). BMAT has been established as a significant source of adiponectin following CR, suggesting that CR-induced endocrine alterations of BMAT could contribute to the beneficial effects of CR on metabolic health (7). In our study, adiponectin expression was elevated in all regions of the tibia upon CR and could therefore potentially mediate the beneficial effects of CR on bone. Moreover, CR, but not age, resulted in increased levels of circulating adiponectin. The literature on the effects of adiponectin on bone health is somewhat inconclusive, as some studies suggested positive effects on bone mass, while other studies reported a negative effect (43). Recently it has been established that binding of the low molecular weight isoform to the respective receptor in osteoblasts increases their differentiation capacity (44). Moreover, agonist-based activation of the adiponectin receptor enhanced osteogenic differentiation of bone stem cells, while inhibiting osteoclast differentiation and activation (45). Adiponectin is a potent inducer of mitochondrial biogenesis and energy metabolism, and it is thus conceivable that this adipokine, which is preferentially secreted from BMAT under CR, is involved in the increased expression of mitochondrial genes in aged mice (46). Little is known about the effects of CR on mitochondrial function and its link to adiponectin, specifically in the bone, but increased mitochondrial biogenesis is well established and described in many tissues, including the brain, liver, heart, and brown adipose tissue upon CR (47). Moreover, CR restored mitochondrial DNA content in muscle and liver in aged mice (48). These results suggest that CR might restore age-related loss of mitochondrial function in a process involving elevated secretion of adiponectin from BMAT and our results provide evidence for such a mechanism occurring in the long bone.

In humans, most data on BMAT accumulation during weight loss derive from patients suffering from Anorexia nervosa. This eating disorder features severe restriction of caloric intake and is linked to high BMAT content, low bone mineral density, and higher risk for osteoporosis and fractures (16). However, the weight loss in Anorexia nervosa patients could be more extreme and is accompanied by other aspects of malnutrition, including micronutrient deficiencies and hormonal perturbations, which could all negatively affect bone health (49). However, a recent study established that fasting for 10 days can also lead to a significant increase in BMAT accumulation in humans, suggesting that BMAT plays an important role even during short-term energy depletion (18).While the negative effects of Anorexia nervosa on the bone are clear, classical weight loss interventions in overweight individuals are less harmful to bone health. The extent and timing of CR appears to be critical when trying to maintain bone mass. Hip bone mineral density decreases when an extreme weight loss protocol of 65–75% restriction was performed. With a milder approach of 25–35%, hip bone mineral density was preserved (50). The negative effects of CR on bone quality might be ameliorated when micronutrient supply is adequate. When CR is paired with micronutrient enrichment, the reduction in bone mineral density is not accompanied by reduced bone quality and microstructure (51). Calcium intake and physical exercise during CR appear to be particularly effective to minimize detrimental effects on bone health (52, 53).

While our study shows the positive effect of CR on aged bone, our study layout has some potential limitations. Studies were only performed in male mice. Given that many aspects of bone biology display sexual dimorphisms, future studies are needed to determine whether similar effects occur during CR in female mice. Secondly, 15–18 months old mice were assigned to the aged group. While we believe that the results from **Figures 2**, **3** illustrate a robust detrimental effect of this age on non-fractured bone, other studies on bone aging have used mice of different age groups, mainly 12 or 24 months of age (37, 39, 54), making it difficult to compare our data directly to other studies. A technical issue that should be mentioned in our study is that bone length could be slightly different between the young and the aged groups. This could result in differences in the areas analyzed by micro-CT, which then span slightly different percentages of the bone in aged compared to young mice.

Taken together, our data indicate that caloric restriction and aging both induce a marked accumulation of BMAT while exerting distinct effects on bone health. Our findings suggest that the endocrine profile of BMAT may be a key element that renders marrow-resident adipocytes a beneficial or detrimental factor in the maintenance of bone health. This may be partly mediated by the adipokine profile of BMAT, where high expression of adiponectin could drive a mechanism of restored mitochondrial metabolism and improved long-bone quality in aged mice. Conversely, the literature provides evidence for the notion that extreme and uncontrolled weight loss is damaging to the bone, potentially by introducing a combination of micronutrient deficiency and excessive BMAT accumulation with pathological endocrine properties. While moderate CR paired with micronutrient supplementation could prevent the negative effects on the bone, the beneficial aspects of CR on endocrine function of BMAT and bone health are maintained.

## Data availability statement

The data presented in the study are deposited in the Gene Expression Omnibus (GEO) repository, accession number GSE262441.

## Ethics statement

The animal study was approved by State Office of Environment, Health, and Consumer Protection, State of Brandenburg, Germany. The study was conducted in accordance with the local legislation and institutional requirements.

## Author contributions

CR: Conceptualization, Formal analysis, Investigation, Methodology, Validation, Visualization, Writing – original draft, Writing – review & editing. GS: Formal analysis, Validation, Writing – review & editing. MO: Formal analysis, Writing – review & editing. ML: Formal analysis, Writing – review & editing. OS-B: Writing – review & editing, Methodology, Resources. L-MB: Methodology, Writing – review & editing. CB: Methodology, Writing – review & editing. EA: Methodology, Writing – review & editing, Formal analysis. MM: Formal analysis, Methodology, Writing – review & editing. GD: Methodology, Writing – review & editing, Resources, Validation. LS: Methodology, Resources, Writing – review & editing, Data curation, Formal analysis. KS-B: Data curation, Formal analysis, Methodology, Resources, Writing – review & editing, Conceptualization, Funding acquisition, Investigation, Writing – original draft. TS: Conceptualization, Data curation, Formal analysis, Funding acquisition, Investigation, Methodology, Resources, Writing – original draft, Writing – review & editing, Project administration, Supervision, Visualization.
