# Supplementary material for: Caloric restriction reduces trabecular bone loss during aging and improves bone marrow adipocyte endocrine function in male mice
Source: Front Endocrinol (Lausanne). 2024 Jun 5;15:1394263. doi: 10.3389/fendo.2024.1394263 (PMC11188307; doi:10.3389/fendo.2024.1394263)
Supplement: Supplementary file 1 [file DataSheet_1.pdf]

Supplementary Material

Supplementary Figure S1

Experimental layout of the dietary interventions, animal ages at start / end of experiment, and information on the analyses performed on the mice.

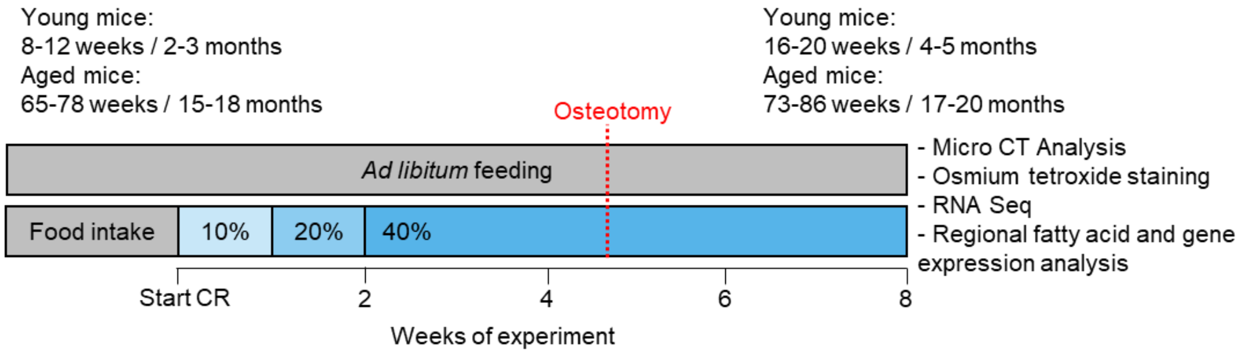

Supplementary Figure S2

3D-renders of the analyzed trabecular regions in cross-sectional orientation. Scanned regions from all individual mice in each diet/age group are displayed.

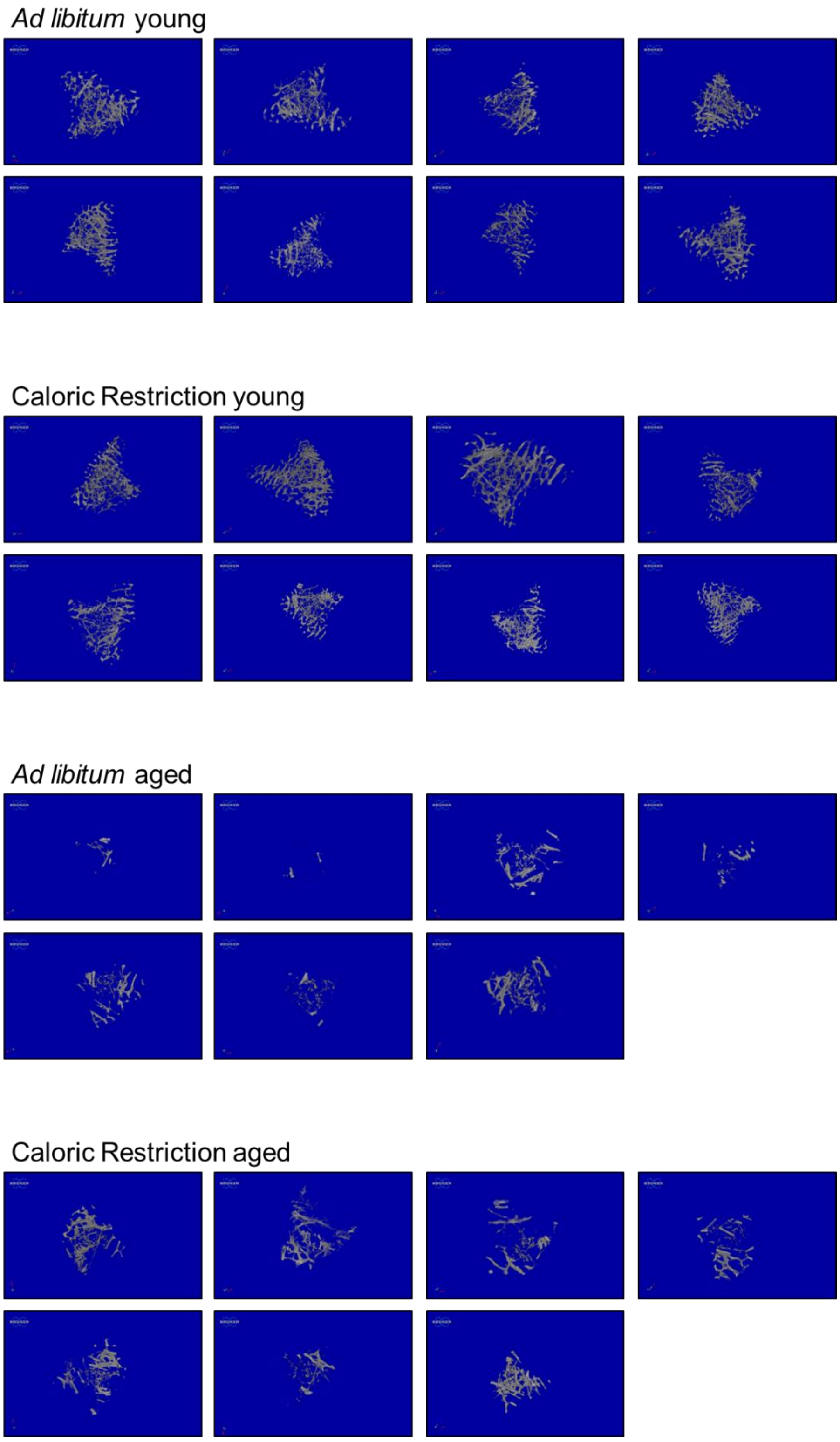

Supplementary Figure S3

3D-renders of the analyzed trabecular regions in longitudinal orientation. Scanned regions from all individual mice in each diet/age group are displayed.

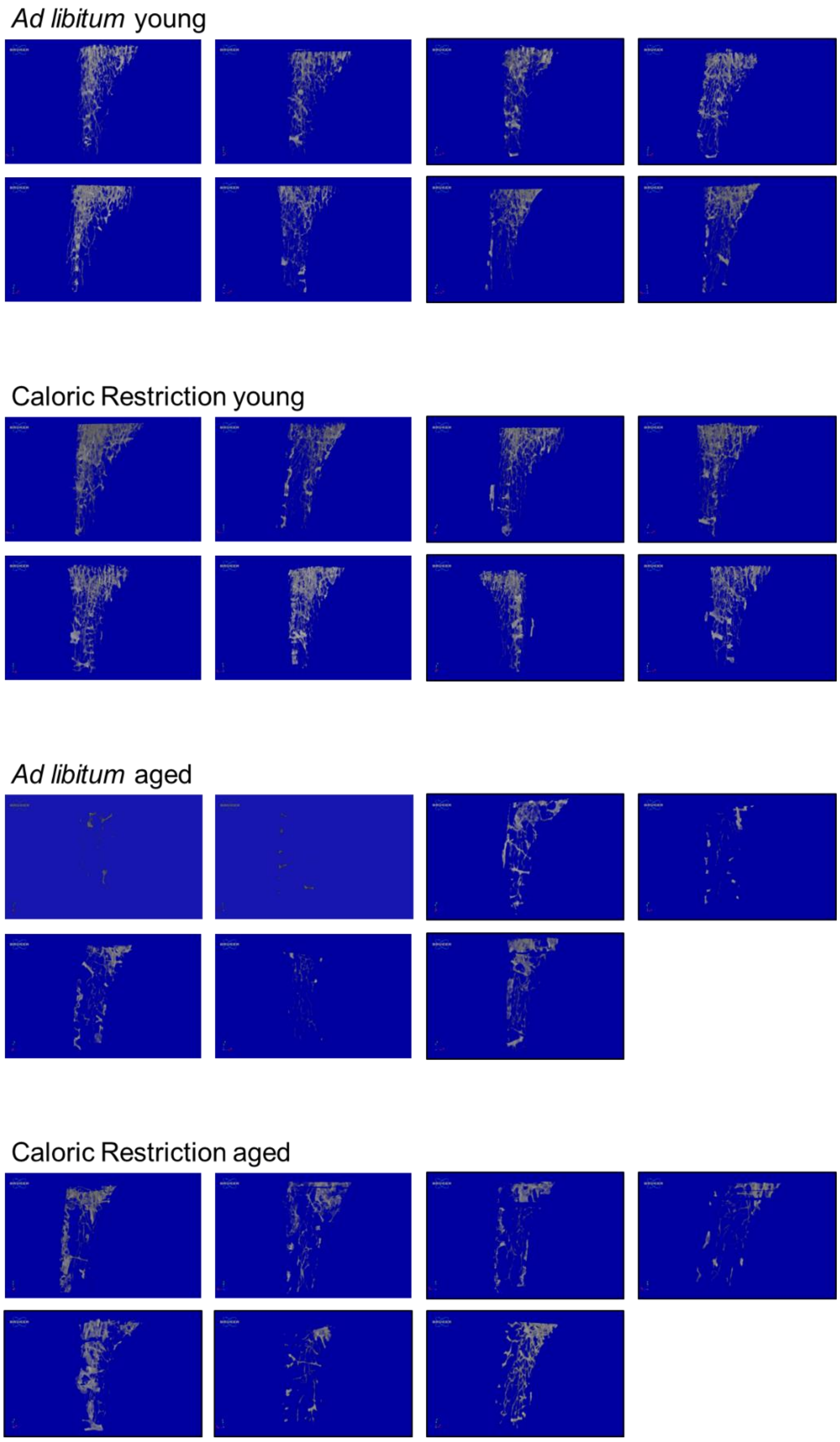

Supplementary Figure S4

Cross-sectional 3D-rendered images of the analyzed cortical bone region. Scanned regions from all individual mice in each diet/age group are displayed.

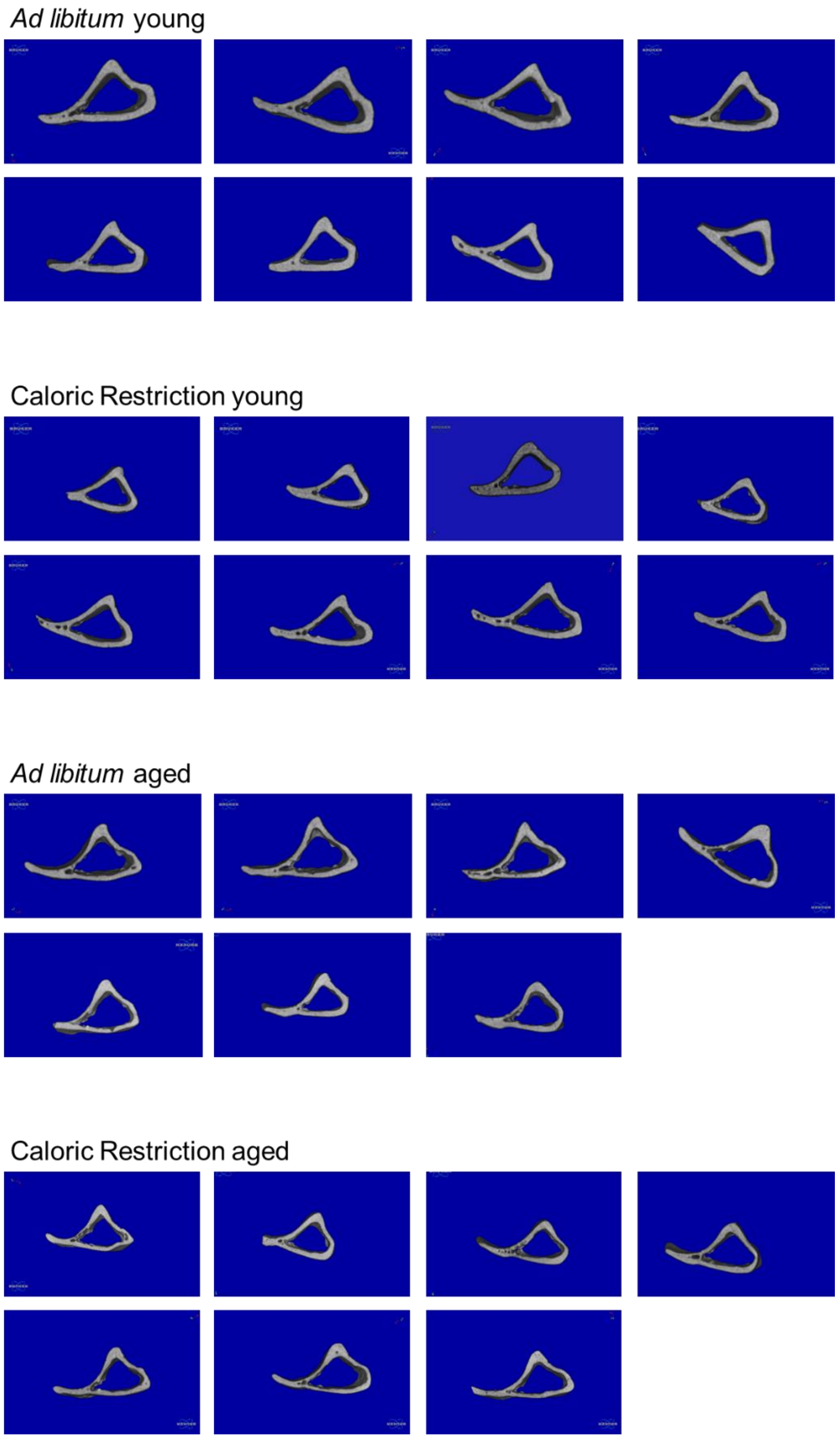

Supplementary Table 1

Primer sequences used in this study.

Supplementary Table 2

Fatty acid profiles in tibiae that were subdivided into three regions prior to analysis, representing the proximal, diaphyseal, and distal BMAT volumes. Fatty acid profiles were recorded in young ad libitum fed (AL) control mice (n=4), young mice on CR (n=4), aged ad libitum fed mice (n=4), and aged mice on CR (n=3).
