# Supplementary material for: Caloric restriction reduces trabecular bone loss during aging and improves bone marrow adipocyte endocrine function in male mice
Source: Front Endocrinol (Lausanne). 2024 Jun 5;15:1394263. doi: 10.3389/fendo.2024.1394263 (PMC11188307; doi:10.3389/fendo.2024.1394263)
Supplement: Supplementary file 2 [file Table_1.docx]

**Supplementary Table 1. Primer Sequences**

| Gene | Sequence (5’🡪3’) |
| --- | --- |
| *Adipoq* | FWD: GGCAGGAAAGGAGAACCTGG  REV: AGCCTTGTCCCTCTTGAAGAG |
| *Plin1* | FWD: CTGTGTGCAATGCCTATGAGA  REV: CTGGAGGGTATTGAAGAGCCG |
| *Scd1* | FWD: CAGGTTTCCAAGCGCAGTTC  REV: ACTGGAGATCTCTTGGAGCA |
| *Tbp* | FWD: ACGCTTCACCAATGACTCCTA  REV: TGACTGCAGCAAATCGCTTGG |
